# Supplementary material for: Personal Genomes in Practice: Exploring Citizen and Healthcare Professionals’ Perspectives on Personalized Genomic Medicine and Personal Health Data Spaces Using a Mixed-Methods Design
Source: Genes (Basel). 2023 Mar 24;14(4):786. doi: 10.3390/genes14040786 (PMC10137790; doi:10.3390/genes14040786)
Supplement: Supplementary file 1 [file genes-14-00786-s001.zip › Interview Protocol PGL Focus groups_healthcare professionals.pdf]

Gesprekshandleiding focusgroepen PGL – burgers/patiënten

Inleiding 10-15 min.

Welkom
Goedemorgen allemaal, welkom bij de focusgroepsessie. Dank jullie wel voor jullie interesse en dat jullie tijd vrij hebben gemaakt om deel te nemen. Kunnen jullie mij allemaal zien en horen?

Mijn naam is [NAAM] en ik werk als onderzoeker bij de afdeling Gezondheids, Medische en Neuropsychologie van de Universiteit Leiden. Ik wil jullie ook graag nog even voorstellen aan [NAAM]. [NAAM] is hier vandaag als observator en zal aantekeningen maken van het gesprek. [NAAM] kun je jezelf even voorstellen?

We zijn hier vandaag om te praten persoonlijke DNA-datakluis. Misschien is niet iedereen hier even bekend mee en dat is ook niet erg. Voordat we beginnen met de discussie zal ik het onderwerp eerst wat meer toe lichten .

Praktische zaken
Voordat we daadwerkelijk het gesprek gaan beginnen, wil ik graag enkele praktische zaken met u doornemen:

- *[spelregels in PowerPoint-dia]* We zullen alles wat vandaag besproken wordt opnemen (alleen geluid) zodat we dit later heel goed kunnen naluisteren en geen dingen vergeten. Ikzelf en [naam observator] zullen aantekeningen maken. Bent u daarmee akkoord? Dan start nu de opname.
- Alles wat gezegd wordt is vertrouwelijk en er zullen geen namen verschijnen in de rapportage van het onderzoek. Dus voelt u zich vrij om uw mening te delen.
- Er zijn dan ook geen goede of foute antwoorden.
- Het is niet erg om het niet met elkaar eens te zijn. Zolang we maar respectvol naar elkaar luisteren, is het goed.
- Ik ben hier om het gesprek te leiden. Ik zal vragen stellen en ik zal ervoor zorgen dat iedereen aan het woord komt. Verder is het de bedoeling dat jullie vooral met elkaar in gesprek gaan. Ga dus vooral op elkaar in, stel vragen of geef voorbeelden.
- Omdat we deze focusgroep digitaal houden, kan er maar 1 persoon tegelijk praten. Zou u uw microfoon uit willen zetten als u niet aan het woord bent, om teveel achtergrondgeluid te voorkomen? *[screenshot functionaliteit microfoon]*. Voelt u zich vrij om uw geluid weer aan te zetten als u iets wilt zeggen. U mag eventueel ook uw hand opsteken als u het woord wilt.
- Het is het meest persoonlijk als u uw camera aan laat staan gedurende het gesprek *[screenshot functionaliteit beeld]*. U kunt iedereen zien als u gebruik maakt van de 'gallery view'*[screenshot gallery view]*. Lukt dat bij iedereen?
- Soms zal ik u vragen om een vraag eerst in de chat te beantwoorden *[screenshot functionaliteit chat]*. Ziet u de chatfunctie?
- Ik zal af en toe meeschrijven met de antwoorden die worden gegeven, met behulp van het bord, zodat het voor iedereen zichtbaar is.
- De discussie van vandaag zal ongeveer 2 uur duren. We zullen 10-15 minuten pauze houden, voelt u zich tussendoor ook vrij om even op te staan als dat nodig is, bijvoorbeeld om iets te drinken te pakken of om naar het toilet te gaan. Is dat voor iedereen oké?
- Ik zou willen vragen om telefoons uit of op stil te zetten. Zit iedereen in een rustige ruimte waarin u niet teveel gestoord zult worden en met een goede internetverbinding?

Verder is het handig als u pen en papier bij de hand hebt, en uw antwoorden op de vragen die we in de mail vooraf hebben gestuurd.
- Mocht u tussendoor technische problemen krijgen, dan kunt u contact opnemen met *[naam observator]* via *[telefoonnummer]* of *[mail]*.
- Zijn er nog vragen voordat we beginnen?

Planning
Dan zal ik nu de planning voor vandaag even toelichten *[planning PowerPoint]:*

- Voorstellen
- Toelichting DNA-datakluis
- Discussie: toegang tot medische gegevens, DNA-onderzoek, DNA-datakluis
- Afronding

Voorstellen
[PowerPoint voorstelrondje] Maar laten we eerst beginnen met een kort voorstelrondje. Zou iedereen even kort zijn naam willen noemen, je werk, en wat je motivatie is geweest om mee te praten vandaag.

Introductie DNA-datakluizen tijd

1. Wat stelt u zich voor bij een DNA-datakluis? Schrijf een eerste reactie in de chat. *In gesprek n.a.v. reacties. Daarna definitie DNA-datakluis:*

   [PowerPoint DNA-datakluis] De DNA-datakluis is een persoonlijke, beveiligde omgeving waarin mensen hun DNA-profielen kunnen opslaan, bekijken en interpreteren. Ook kunnen mensen hun DNA-profiel delen met zorgverleners en onderzoekers. Zorgverleners kunnen het DNA-profiel vervolgens gebruiken bij het voorschrijven van medicatie maar ook bij preventie.

   Een voorbeeld waarbij zo'n DNA-datakluis behulpzaam zou kunnen zijn is bij genetische aandoeningen. Een voorbeeld hiervan is de spierziekte Duchenne spierdystrofie. *[PowerPoint Duchenne]*

   Casus beschrijving Duchenne spierdystrofie
   Duchenne spierdystrofie is een ernstige erfelijke spierziekte die de spieren aantast en verzwakt. De eerste verschijnselen zijn vaak al voor het tweede levensjaar zichtbaar. Op den duur kunnen de aangetaste spieren niet meer gebruikt worden.
   Duchenne spierdystrofie treft nagenoeg altijd jongens. Meisjes kunnen echter wel drager zijn van het gen en het doorgeven aan hun kinderen.

   Met DNA-onderzoek kan in veel gevallen een afwijking in het dystrofine-gen worden aangetoond. Meisjes worden echter niet standaard getest. Je moet daar dus zelf aan denken. En je moet dan wel weten dat Duchenne in je familie voorkomt.

   Wat zou helpen is als je al die informatie, inclusief je familiegeschiedenis, op één plek zou kunnen bewaren. Zodat je ook geen problemen hebt als je van huisarts wisselt of verschillende zorgverleners hebt. En dat je bijvoorbeeld op je 18e een melding krijgt dat je

mogelijk drager bent en dat je je kunt laten testen als je dat wilt. Hier zou een DNA-datakluis in kunnen voorzien.

Oefenen met demo
Om jullie iets meer een idee te geven hoe een DNA-datakluis eruit zou kunnen zien, gaan we nu oefenen met een demo.
In dit voorbeeld hebben we zogenaamd een DNA-test gedaan bij het LUMC. Nu vraagt onze huisarts om toestemming om deze gegevens in te zien.
Script beschrijven.

Toegang tot medische gegevens tijd

2. Wie vertrouw je met je (medische) gegevens van patiënten? Schrijf een eerste reactie in de chat. *In gesprek n.a.v. reacties.*

3. Wie moet de controle hebben over de gegevens? Oftewel: wie zou de baas moeten zijn over de gegevens? Schrijf een eerste reactie in de chat. *In gesprek n.a.v. reacties.*

   Huisarts
   Ziekenhuis
   Patiënt zelf

   Patiënt heeft de controle:
   Voordelen en nadelen? (verstand van zaken, mondigheid, empowerment)

   Huisarts heeft de controle:
   Voordelen en nadelen? (namens jou, kennis, 1e contact)
   Is dat wenselijk? Is hier draagvlak voor?

4. Als we het hebben over het delen van medische gegevens met zorgverleners. Wat zouden dan de voordelen zijn? Schrijf een eerste reactie in de chat. *In gesprek n.a.v. reacties.*

   Voordelen? (efficiënte zorg, zorgverleners kunnen er snel bij, medische kwetsbaarheid, onderzoeksdoeleinden)

5. En de nadelen?

   Nadelen: schade door privacy verlies. Sociale kwetsbaarheid.

[PAUZE?]

De Personal Genetic Locker tijd

We hebben het nu gehad over de toegang tot medische gegevens.

Dan gaan we het nu hebben over de Personal Genetic Locker, dit is de DNA-datakluis waarin je je DNA-profiel kunt opslaan, bekijken en interpreteren en kunt met zorgverleners en onderzoekers.

6. Of je wilt weten of je een erfelijke aandoening hebt of kunt krijgen, is persoonlijk. Hoe moeten we hier in de DNA-datakluis mee omgaan? Poll
   - De patiënt geeft voorafgaand aan het DNA-onderzoek wat wel en niet onderzocht mag worden → resultaat: zowel patiënt als zorgverlener ontvangen alleen informatie over de analyses waar de patiënt toestemming voor heeft gegeven.
     a. Zou het aangeven van waar wel en niet naar gekeken mag worden iets eenmaligs zijn of moet je dat om de zoveel tijd vragen? Bijvoorbeeld als er nieuwe kennis/behandelmogelijkheden ontstaan?
   - De patiënt geeft voorafgaand aan het DNA-onderzoek aan wat hij/zij wel of niet wil weten. → resultaat: een zorgverlener weet soms meer dan een patiënt

7. Zou u er zelf voor kiezen om DNA-datakluizen te implementeren in de zorg? Waarom wel? Waarom niet? Poll. *In gesprek n.a.v. reacties.*

8. Wat zou de implementatie kunnen belemmeren?

9. Zou je voor een DNA-datakluis willen betalen? Of wie zou er anders voor moeten betalen? Poll. *In gesprek n.a.v. reacties. Deze niet?*

Afronding tijd

We hebben vandaag veel besproken en er zijn veel verschillende ideeën en meningen langsgekomen. We zijn benieuwd wat ieder van u het meest is bijgebleven, daarom willen we nog een rondje doen:

- Noem 1 ding wat u het meest is bijgebleven van dit gesprek, wat u ons als onderzoekers zeker zou willen meegeven over persoonlijke DNA-datakluizen.
- Heeft iemand nog vragen of andere aanvullingen naar aanleiding van wat we vandaag hebben besproken?

Ik wil u nogmaals heel erg bedanken voor uw komst en deelname vandaag. Wij gaan alles wat u ons vandaag heeft verteld zorgvuldig verwerken. Tot slot wil ik graag nog een laatste vraag stellen:

Alvast heel erg bedankt hiervoor.

Mocht u nog vragen hebben, dan kunt u natuurlijk altijd contact opnemen!
